# Metabolic syndrome among pre- and post-menopausal rural women in Bangladesh: result from a population-based study

**DOI:** 10.1186/1756-0500-6-157

**Published:** 2013-04-18

**Authors:** Subrina Jesmin, AM Shahidul Islam, Shamima Akter, Md Majedul Islam, Sayeeda Nusrat Sultana, Naoto Yamaguchi, Osamu Okazaki, Masao Moroi, Michiaki Hiroe, Sosuke Kimura, Tetsu Watanabe, Kawano Saturo, Taro Mizutani

**Affiliations:** 1 Health & Disease Research Center for Rural Peoples (HDRCRP), 14/15, 1st Floor, Probal Housing Ltd., Shekertak (Adjacent to Shekertak Road 1), Mohammadpur, Dhaka, 1207, Bangladesh; 2 National Center for Global Health and Medicine (NCGM), 1-21-1 Toyama, Shinjuku-ku, Tokyo, 162-8655, Japan; 3 Department of Public Health, Tokai University Graduate School of Medicine, Isehara, Japan; 4 Graduate School of Medicine, Faculty of Medicine, University of Tsukuba, Tsukuba, Ibaraki, 305-8575, Japan

**Keywords:** Metabolic syndrome, Pre-menopausal women, Post-menopausal women, Bangladesh

## Abstract

**Background:**

Prevalence of non-communicable diseases are a challenging problems among menopausal women specially in a least developed country like Bangladesh, where majority of women suffering from at least one chronic diseases after menopausal age. So, the main objective of this study was to determine the prevalence of metabolic syndrome and related risk factors in Bangladeshi pre- and post-menopausal women living in the rural setting.

**Methods:**

This study is based on a community based cross-sectional survey among 1802 rural women aged ≥15 years. Metabolic syndrome was defined according to the criteria of NCEP-ATP III. Logistic regression was used to estimate the association between menopausal status and metabolic syndrome and its components.

**Results:**

Metabolic syndrome was presented in 25.6% respondents and it was more prevalent among post-menopausal (39.3%) as compared to pre-menopausal (16.8%) women. Logistic regression analysis reveals that prevalence of metabolic syndrome was 1.78 times higher in post-menopausal women than pre-menopausal women (*P* = 0.001). Prevalence of high blood pressure, elevated fasting blood glucose, and high triglyceride were significantly higher in post-menopausal women than pre-menopausal women (*P* < 0.05). However, prevalence of low high-density lipoprotein cholesterol was significantly lower in post-menopausal women than pre-menopausal women (*P* < 0.001).

**Conclusions:**

Metabolic syndrome seems to be a major health problem among post-menopausal women in many developing countries like Bangladesh and proper policy emphasis should be given on its prevention and control.

## Background

The prevalence of chronic non-communicable diseases such as cardiovascular diseases and type 2 diabetes is increasing significantly in Bangladesh [1]. This increase is observed not only in the urban areas but also in the rural population, especially rural women in Bangladesh seems to be more vulnerable who are mostly poor and physically active [2]. The metabolic syndrome (MS) is a cluster of risk factors including obesity, glucose intolerance, dyslipidemia, and hypertension that increase the risk for cardiovascular disease and type 2 diabetes [3,4]. A striking increase in the number of people with the MS has taken place worldwide not only in developed countries but also in developing countries considerably due to changing environment and lifestyle [5].

Previous studies have showed that MS and cardiovascular diseases are more common in women above 55 years of age with significant increase in individual risk factors in the postmenopausal phase [6,7]. Changing hormonal milieu with decreasing estrogen and alteration of its ratio with testosterone has been implicated as a causal factor for the emergence of MS at menopausal transition [8,9]. Besides menopausal hormonal changes, aging also play roles to clustering of cardio-metabolic risk factors [10]. Questions remain whether menopause has a causative contribution to the deteriorating metabolic profile that is independent of chronological aging.

Prevalence of MS among pre- and post-menopausal women has varied according to different population. To date, several previous studies found a significance difference in prevalence of MS among pre- and post-menopausal women [11-17]. However, studies related to MS and menopausal status is limited in South Asia [14]. South Asian women, in general are prone to have MS at a younger age and have severe morbidity and mortality consequences as compared to Caucasians and other Asians [18,19]. Several studies found that menopausal status is influentially linked to some features of MS [11,14,16]. However, data on the empirical study of MS in Bangladesh are very rare. So far no studies have been conducted in Bangladesh to determine the prevalence of MS among pre- and post- menopausal women who are mostly poor and have limited or no access to health care services. In our previous studies we found that prevalence of metabolic syndrome was nearly 31% among rural women in Bangladesh, more than 85% rural women have low HDL cholesterol and nearly 31% have high fasting blood glucose [20,21]. Thus, it becomes necessary to measure prevalence of MS in Bangladeshi rural women focusing menopausal status for taking an early lifestyle interventions and treatment to prevent many non-communicable diseases in rural population as they are now confronting with an emerging health epidemic of MS. Thus, the objective of this study is to estimate the prevalence of MS in Bangladeshi rural women considering their menopausal status and further examined the association between menopausal status and MS and its components.

## Methods

### Study procedure and subjects

In a community based cross-sectional study of rural women, a total of 1802 participants aged ≥15 years were selected using stratified multistage random sampling. This sample size was sufficient to test all the research hypotheses at the 5% level of significance with a power of 80% (β = 0.20). Subjects were excluded having chronic illness such as hypothyroidism, pregnant women, those on hormone replacement therapy (HRT) as well as women with known illness such as ischaemic heart disease (IHD), diabetes and hypertension. The study uses the World Health Organization’s STEPS approach (modified) which entails stepwise collection of the risk factor data based on standardized questionnaires covering demographic characteristics, somatic illnesses, somatic and mental symptoms, medications, life style, and health-related behavior (step 1), basic physical measures (step 2) and basic biochemical investigations such as blood glucose and cholesterol (step 3). Women were defined as post-menopausal if they had reported their last menses to be at least 12 months previously; pre-menopausal if they had an unchanged and regular menstrual pattern during the last five years, without typical climacteric complaints; and peri-menopausal if last menses to be within or before 12 months and were not regular. In our study we excluded peri-menopausal women. A standard questionnaire on health and menopausal status written in the local language was used to identify the pre- and post-menopausal subjects.

The study was carried out in 4 village communities of Gabindagonj*Upazilla* (subdistrict) of Gaibandha district, a poor district of the northern division of Rajshahi, the poorest region of the country. The respondents were selected randomly after selecting division, district, *Upazila* and villages. Women were recruited through local announcement (loudspeaker) at community level and surveyors visits. The details of the study procedure have been described before in our previous study [21,22]. In brief, for getting required information, the participants were interviewed and examined clinically at mobile examination centre and asked to provide a blood sample. The study was approved by the Ethical Committee of the Health and Disease Research Center of Rural Peoples (HDRCRP), Dhaka, Bangladesh. This study conformed to the principles outlined in the Helsinki Declaration, and all subjects gave their written informed consent before inclusion in the study.

### Anthropometric measurements

Anthropometric measurements on individuals wearing light clothing and without shoes were conducted by well-trained examiners. Height was measured to the nearest 0.1 cm using the portable stadiometer. Weight was measured in the upright position to the nearest 0.1 kg using a calibrated balance beam scale. Body mass index (BMI) was calculated by dividing weight (kg) by height squared (m^2^). Waist circumference measurements were taken at the end of normal expiration to the nearest 0.1 cm, measuring from the narrowest point between the lower borders of the rib cage and the iliac crest. Blood pressure was measured twice in the right arm in a sitting position using the standard mercury manometer and cuff, to the nearest 2 mmHg, with the initial reading taken at least 5 minutes after the subject was made comfortable, and again after an interval of 15 minutes. The average systolic and diastolic blood pressures were then estimated.

### Biochemical analysis

Blood for biochemical analysis was obtained from the participants after 10–12 hours of an overnight fast. The blood sample was collected using standard blood sample collection procedure. After venipuncture of blood the processed serum/plasma were immediately stored at HDRCRP main or sub-center offices. The serum was immediately separated by centrifugation, and the concentration of triglycerides [lipoprotein lipase method; Wako Chemicals, Tokyo, Japan], total cholesterol [Cholesterol E, Wako Pure Chemical Industries, Ltd. Osaka, Japan] and its fractions high-density lipoprotein (HDL) cholesterol [HDL cholesterol with the Determiner-L kit (Kyowa Co Ltd, Tokyo, Japan)] were ascertained. Fasting plasma glucose [glucose with the Hexokinase G-6-PDH kit (Wako Pure Chemical Industries Ltd, Osaka, Japan) were also measured. After labeling blood sample vials, a part of blood were transported to National Centre for Global Health and Medicine (NCGM), Japan for advanced biochemical assessment processing.

### Definition of metabolic syndrome and risk factors

We defined the metabolic syndrome (MS) using the National Cholesterol Education Program Adult Treatment Panel III (NCEP-ATP III) [23]. As detailed in the NCEP-ATP III report, participants having three or more of the five following criteria were defined as having the MS: high blood pressure (≥130/≥85 mmHg), elevated fasting blood glucose (≥110 mg/dL or ≥ 6.1 mmol/L), high triglyceride (≥150 mg/dL or ≥1.65 mmol/L), low high-density lipoprotein (HDL) cholesterol (<50 mg/dL or <1.30 mmol/L), and abdominal obesity, as measured by a waist circumference of ≥88 cm for women. Participants who currently reported using antihypertensive or antidiabetic medication (insulin or oral agents) were counted as having high blood pressure or elevated fasting blood glucose, respectively.

### Statistical analyses

The clinical and laboratory characteristic of study population are presented according to pre- and post-menopausal women. The significant differences between pre- and post-menopausal women were assessed by using the Student’s “t” test for continuous variables and chi-square (χ^2^) test for categorical variables. Logistic regression analyses were used to assess the association between menopausal status and MS and each of its components. Two types of models were used. The first model was crude model without any adjustment and the second model was adjusted for age. Statistical significance was set at the conventional *P* < 0.05. All analysis were performed using statistical software STATA version 12.0 (Lakeway Drive, College Station, Texas USA).

## Result

**Figure 1:**
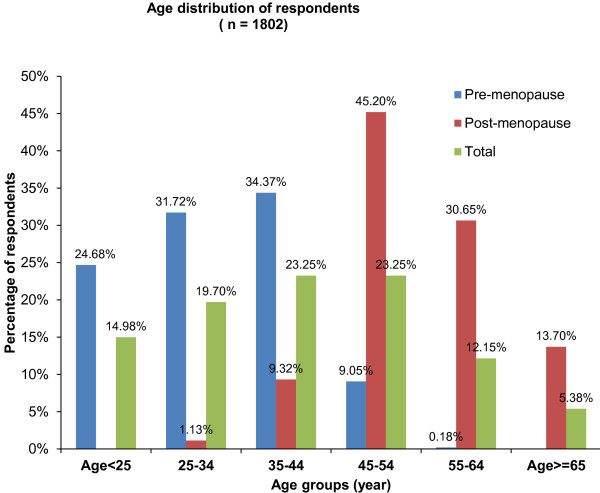
Age distribution of respondents by menopausal status.

The clinical and laboratory characteristics of the study population stratified by menopausal status and MS are presented in Table 1. A total of 1802 subjects turned out for data analysis. There were 1094 pre-menopausal and 708 post-menopausal women (ratio 1:1.55); and their ages ranged from 15–85 (mean 39.98 ± 13.97) years. Figure 1 shows the age and menopausal status distribution of the study subjects. Most (45.20%) of the study subjects were in the 45–54 year age group. There were more pre-menopausal than post-menopausal women across all age groups. The pre-menopausal women were aged 15–51 years (mean 31.36 ± 9.24) while post-menopausal women were aged 32–85 years (mean 53.31 ± 8.49) (*P* < 0.001).

**Table 1:** Means of clinical and laboratory characteristics of the subjects

**Variable**	**Overall**	**Pre-menopause**	**Post-menopause**	**P value**
	**(n = 1802)**	**(n = 1094)**	**(n = 708)**	
Age, years	39.98 ± 13.97	31.36 ± 9.24	53.31 ± 8.49	<0.001
BMI, kg/m^2^	21.88 ± 3.97	21.87 ± 3.99	21.89 ± 3.94	0.95
Waist circumference, cm	76.83 ± 8.32	76.89 ± 8.54	76.73 ± 7.98	<0.001
Systolic blood pressure, mmHg	116.93 ± 21.38	110.56 ± 17.95	126 ± 22.51	<0.001
Diastolic blood pressure, mmHg	75.53 ± 10.78	73.00 ± 9.80	79.44 ± 11.07	<0.001
Fasting blood glucose, mmol/L	6.22 ± 2.72	5.77 ± 2.12	6.91 ± 3.35	<0.001
Total cholesterol, mg/dL	181.09 ± 72.32	176.15 ± 70.00	188.75 ± 75.18	<0.001
HDL cholesterol, mg/dL	41.38 ± 30.47	37.34 ± 17.69	45.69 ± 43.20	<0.001
Triglyceride, mg/dL	130.02 ± 109.95	114.79 ± 92.64	153.60 ± 128.94	0.007

Values are Mean ± SD. BMI: Body mass index, HDL cholesterol: High-density lipoprotein cholesterol.

The prevalence of MS and metabolic risk factors are presented in Table 2. Prevalence of MS was 25.6% in our study, where 39.3% of post-menopausal women versus 16.8% of pre-menopausal women had MS (*P* < 0.001). Prevalence of metabolic risk factors including elevated fasting blood glucose, high blood pressures, and high triglycerides were significantly higher in post-menopausal women than pre-menopausal women (*P* < 0.001). However, prevalence of low HDL cholesterol was significantly lower in post-menopausal women than pre-menopausal women (*P* = 0.001).

**Table 2:** Frequency of metabolic risk factors and metabolic syndrome

**Risk factors**	**Overall**	**Pre-menopause**	**Post-menopause**	**P value**
	**n (%)**	**n (%)**	**n (%)**	
BMI > 30 kg/m^2^	52 (2.9)	34 (3.11)	18 (2.56)	0.50
Elevated waist circumference	152 (8.46)	91 (8.32)	61 (8.68)	0.79
High blood pressures	506 (28.16)	159 (14.53)	347 (49.36)	<0.001
Elevated fasting blood glucose	636 (35.39)	287 (26.23)	349 (49.64)	<0.001
Low HDL cholesterol	1512 (84.14)	972 (88.85)	540 (76.81)	0.001
High triglyceride	530(29.49)	262 (23.95)	268 (38.12)	<0.001
Metabolic Syndrome ^a^	460 (25.60)	184 (16.82)	276 (39.26)	<0.001

BMI: Body mass index, HDL cholesterol: High-density lipoprotein cholesterol.

^a^ Metabolic syndrome is defined as presence of at least 3 of the following criteria.

Elevated waist circumference (≥88 cm), high fasting blood glucose (≥110 mg/dL), high triglycerides (≥150 mg/dL), low-high-density lipoprotein (HDL) cholesterol (<50 mg/dL), high blood pressure; systolic blood pressure (SBP ≥ 130 mm Hg) or diastolic blood pressure (DBP ≥85 mm Hg).

Table 3 shows odds ratio (OR) and 95% confidence interval (CI) of MS and each component of MS according to menopausal status. In unadjusted model the prevalence of MS was 3.14 times more likely (CI: 2.52-3.92) in post-menopausal women than pre-menopausal women (*P* < 0.001). After adjustment for age the association was slightly attenuated but remains statistically significant (OR: 1.78, CI: 1.26-2.51, *P* = 0.001). Prevalence of elevated fasting blood glucose, high blood pressures, and high triglycerides were also significantly higher but prevalence of low HDL cholesterol were significantly lower in post-menopausal women than pre-menopausal women even after adjustment for age (*P* <0.05 for all).

**Table 3:** Odds ratio (95% Confidence interval) for metabolic syndrome and its components by menopausal status

**Risk factors**	**Pre-menopause**	**Post-menopause**	***P *value**^**a**^
Elevated waist circumference			
Unadjusted	1.00	1.04 (0.74-1.46)	0.829
Age-adjusted	1.00	0.66 (0.39-1.12)	0.124
High blood pressures			
Unadjusted	1.00	5.65 (4.50-7.07)	<0.001
Age-adjusted	1.00	1.72 (1.22-2.43)	0.002
Elevated fasting blood glucose			
Unadjusted	1.00	2.84 (2.32-3.47)	<0.001
Age-adjusted	1.00	1.63 (1.19-2.23)	0.002
Low HDL cholesterol			
Unadjusted	1.00	0.41 (0.31-0.53)	<0.001
Age-adjusted	1.00	0.44 (0.29-0.67)	<0.001
High triglyceride			
Unadjusted	1.00	1.96 (1.59-2.41)	<0.001
Age-adjusted	1.00	2.27 (1.62-3.17)	<0.001
Metabolic syndrome ^b^			
Unadjusted	1.00	3.14 (2.52-3.92)	<0.001
Age-adjusted	1.00	1.78 (1.26-2.51)	0.001

HDL cholesterol: High-density lipoprotein cholesterol.

^a^ Based on logistic regression analysis.

^b^ Metabolic syndrome is defined as presence of at least 3 of the following criteria.

Elevated waist circumference (≥88 cm), high fasting blood glucose (≥110 mg/dL), high triglycerides (≥150 mg/dL), low-high-density lipoprotein (HDL) cholesterol (<50 mg/dL), high blood pressure; systolic blood pressure (SBP ≥ 130 mm Hg) or diastolic blood pressure (DBP ≥85 mm Hg).

## Discussion

In this cross-sectional study of Bangladesh we compare prevalence of MS and risk variables of MS in pre- and post-menopausal women and also identified how these variables relate to one another. This is the first report in Bangladesh investigating the prevalence of MS and each component of MS risk factors among rural women according to their menopausal status.

The prevalence of MS was higher in post-menopausal (39.3%) women than pre-menopausal (16.8%) women in our study and this prevalence was more likely in post-menopausal women even after adjustment for age. Our findings are consistent with many of previous studies [11,13-16,24], where post-menopausal women have been found to be at higher risk of MS than pre-menopausal women. Ruan et al., found that the prevalence of MS was 34% among Chinese post-menopausal women [24]. According to a study of Western India the prevalence of MS was 45% among pre-menopausal women, whereas it was 55% among post-menopausal women [14]. In another study of India, a prevalence of 22.2% in the pre-menopausal as compared to 32.2% in the post-menopausal group was reported [25]. Arguing menopause as an independent predictor of MS, Eshtiaghi et al., showed a prevalence of 53.5% MS in post-menopausal Iranian women, on the other hand it was only 18% in pre-menopausal women [13]. Taken together, post-menopausal women are more prone to have MS than pre-menopausal women in Bangladesh.

The transition from pre- to post-menopause may be associated with each feature of the MS. In our study metabolic risk factors including high blood pressures, high triglyceride, and elevated fasting blood glucose are significantly higher among post-menopausal women than pre-menopausal women. However, prevalence of low HDL cholesterol is significantly lower in post-menopausal women than pre-menopausal women. In agreement with the results of our study, many previous studies have reported higher concentrations of hypertension [11,15,26], hypercholesterolemia [15], hypertriglyceridemia [5], and elevated fasting blood glucose [11,15] among post-menopausal women than pre-menopausal women. The decreased prevalence of low HDL cholesterol among post-menopausal women than pre-menopausal women is supported by a previous study [27]. Nonetheless, lower prevalence of low HDL cholesterol among post-menopausal women than pre-menopausal women is at variance with other studies [11,15]. Further research is required to elucidate the inconsistencies among studies.

The emergence of metabolic risk factors in post-menopausal phase may be a direct result of ovarian failure with estrogen deficiency. Decrease in estrogen production is thought to be responsible for substantial proportion of increased cardio-metabolic risk factors in post-menopausal women. Changes in metabolic risk factors during pre- and post-menopause may be due to a combination of factors of the aging process. Although previous studies showed age as the predominant variable associated with increased prevalence of MS, but higher prevalence of MS among women >50 years compared with men of the same age has been reported [28]. In our study the association between menopausal status and each component of metabolic MS was not significantly changed even after adjustment for age, which implies that post-menopausal women were indeed at higher risk of having MS and cardiovascular diseases.

In our study we found no significant difference in obesity component including BMI and waist circumference. Our findings are consistent with two previous studies of India [14] and Iran [16]. In addition, in a Framingham study [29] no significant difference was detected between BMI of pre- and post-menopausal women. However, significant differences in central body fat and BMI among pre- and post-menopausal women have been found in many other studies [11,15]. During menopause the pattern of hormone secretion changes and gradually causes fat accumulation in visceral tissues of abdomen and as a result, central obesity. However, the literature to date is not clear as to menopause itself is directly associated with obesity. Results of our study demonstrated that although there was no significant difference in abdominal obesity, the risk of MS was more prevalent among post-menopausal women due to significant difference among other component of MS. Differences in socio-environmental and genetic factors, lifestyles, type of menopause (natural/surgical) could be some of the reasons for this variability.

To the best of our knowledge, our study is the first to demonstrate the prevalence of MS and its components among pre- and post-menopausal women in a low-income country like Bangladesh. One of the major strengths of the present study is that it is based on the largest community-based and comprehensive MS survey. However, our study has several limitations that need to be mentioned. First, an association derived from a cross-sectional study does not necessarily indicate causality. Second, menopausal status was self-reported and could have recall bias. Third, our study may have selection bias during cases recruitment due to selection of rural women from lower socioeconomic class and may not be generalized to the whole Bangladeshi women.

## Conclusions

In conclusion, the results of our study suggest that the prevalence of MS in post-menopausal women was significantly higher than pre-menopausal women. Low HDL cholesterol, elevated fasting blood glucose, and high blood pressures were the most frequent features in comparison to the others. Prevention through changes in lifestyle, or early detection and treatment of elevated fasting blood glucose, hypertension, and hyperlipidemia are necessary for prevention of many chronic diseases in Bangladeshi women reaching after menopausal status. Health professionals should consider the post-menopausal women as a major target group for prevention of MS, which is an underlying condition of many non-communicable diseases.

## Abbreviations

MS: Metabolic syndrome; BMI: Body mass index; HDL cholesterol: High-density lipoprotein cholesterol

## Competing interests

The authors declare that they have no competing interests.

## Authors’ contributions

SJ developed the idea for this paper, wrote the first draft of the manuscript and extensively edited the manuscript; AMSI and SA contributed to statistical analysis and writing results; MMI, and SNS performed all searches and compiled the text; NY, MH, MM and TW provided conceptual and editorial input. All authors equally contributed to discussion and approved the final version of manuscript.

## References

[B1] Bleich SN Koehlmoos TL Rashid M Peters DH Anderson G Noncommunicable chronic disease in Bangladesh: overview of existing programs and priorities going forward Health Policy 2011 100 282 289 10.1016/j.healthpol.2010.09.004 20889225 PMC3043199

[B2] World Health Organization (WHO) Noncommunicable Diseases Country Profiles 2011 Geneva: WHO

[B3] Mottillo S Filion KB Genest J Joseph L Pilote L Poirier P Rinfret S Schiffrin EL Eisenberg MJ The metabolic syndrome and cardiovascular risk: a systematic review and meta-analysis J Am Coll Cardiol 2010 56 1113 1132 10.1016/j.jacc.2010.05.034 20863953

[B4] Ford ES Li C Sattar N Metabolic syndrome and incident diabetes: current state of the evidence Diabetes Care 2008 31 1898 1904 10.2337/dc08-0423 18591398 PMC2518368

[B5] Cameron AJ Shaw JE Zimmet PZ The metabolic syndrome: prevalence in worldwide populations Endocrinol Metab Clin North Am 2004 33 351 375 10.1016/j.ecl.2004.03.005 15158523

[B6] Mesch VR Boero LE Siseles NO Royer M Prada M Sayegh F Schreier L Benencia HJ Berg GA Metabolic syndrome throughout the menopausal transition: influence of age and menopausal status Climacteric 2006 9 40 48 10.1080/13697130500487331 16428124

[B7] Lejsková M Alušík S Suchánek M Zecová S Pitha J Menopause: clustering of metabolic syndrome components and population changes in insulin resistance Climacteric 2011 14 83 91 10.3109/13697131003692745 20443721

[B8] Mesch VR Siseles NO Maidana PN Boero LE Sayegh F Prada M Royer M Schreier L Benencia HJ Berg GA Androgens in relationship to cardiovascular risk factors in the menopausal transition Climacteric 2008 11 509 517 10.1080/13697130802416640 18991078

[B9] Janssen I Powell LH Crawford S Lasley B Sutton-Tyrrell K Menopause and the metabolic syndrome: the Study of Women’s Health Across the Nation Arch Intern Med 2008 168 1568 1575 10.1001/archinte.168.14.1568 18663170 PMC2894539

[B10] Moebus S Balijepalli C Lösch C Göres L von Stritzky B Bramlage P Wasem J Jöckel KH Age- and sex-specific prevalence and ten-year risk for cardiovascular disease of all 16 risk factor combinations of the metabolic syndrome - A cross-sectional study Cardiovasc Diabetol 2010 9 34 10.1186/1475-2840-9-34 20696055 PMC2929217

[B11] Lin WY Yang WS Lee LT Chen CY Liu CS Lin CC Huang KC Insulin resistance, obesity, and metabolic syndrome among non-diabetic pre- and postmenopausal women in North Taiwan Int J Obes 2006 30 912 917 10.1038/sj.ijo.0803240 16432538

[B12] Ainy E Mirmiran P Zahedi Asl S Azizi F Prevalence of metabolic syndrome during menopausal transition Tehranian women: Tehran Lipid and Glucose Study (TLGS) Maturitas 2007 58 150 155 10.1016/j.maturitas.2007.07.002 17768019

[B13] Eshtiaghi R Esteghamati A Nakhjavani M Menopause is an independent predictor of metabolic syndrome in Iranian women Maturitas 2010 65 262 266 10.1016/j.maturitas.2009.11.004 19962253

[B14] Pandey S Srinivas M Agashe S Joshi J Galvankar P Prakasam CP Vaidya R Menopause and metabolic syndrome: A study of 498 urban women from western India J Midlife Health 2010 1 63 69 21716770 10.4103/0976-7800.76214 PMC3122506

[B15] Ebrahimpour P Fakhrzadeh H Heshmat R Ghodsi M Bandarian F Larijani B Metabolic syndrome and menopause: A population-based study Diab Metab Syndr 2010 4 5 9 10.1016/j.dsx.2008.04.014

[B16] Heidari R Sadeghi M Talaei M Rabiei K Mohammadifard N Sarrafzadegan N Metabolic syndrome in menopausal transition: Isfahan Healthy Heart Program, a population based study Diabetol Metab Syndr 2010 2 59 10.1186/1758-5996-2-59 20923542 PMC2958965

[B17] Zivkovic TB Vuksanovic M Jelic MA Stojanovic J Buric B Jojic B Milic N Vujovic S Obesity and metabolic syndrome during the menopause transition in Serbian women Climacteric 2011 14 643 648 10.3109/13697137.2011.569595 21878054

[B18] Misra A Khurana L The metabolic syndrome in South Asians: epidemiology, determinants, and prevention Metab Syndr Relat Disord 2009 7 497 514 10.1089/met.2009.0024 19900153

[B19] Pan WH Yeh WT Weng LC Epidemiology of metabolic syndrome in Asia Asia Pac J Clin Nutr 2008 17 37 42 18296297

[B20] Jesmin S Mia S Islam AM Islam R Sultana SN Zaedi S Yamaguchi N Okazaki O Moroi M Kimura S Hiroe M Prevalence of metabolic syndrome among rural Bangladeshi women Diabetes Res Clin Pract 2012 95 1 e7 e9 10.1016/j.diabres.2011.09.025 22015482

[B21] Jesmin S Islam MR Islam AM Mia MS Sultana SN Zaedi S Yamaguchi N Iwashima Y Hiroe M Watanabe T Comprehensive assessment of metabolic syndrome among rural Bangladeshi women BMC Public Health 2012 12 49 10.1186/1471-2458-12-49 22257743 PMC3293056

[B22] Akter S Jesmin S Islam M Sultana SN Okazaki O Hiroe M Moroi M Mizutani T Association of age at menarche with metabolic syndrome and its components in rural Bangladeshi women Nutr Metab (Lond) 2012 9 1 99 10.1186/1743-7075-9-99 23140264 PMC3541253

[B23] Grundy SM Cleeman JI Daniels SR Donato KA Eckel RH Franklin BA Gordon DJ Krauss RM Savage PJ Smith SC Jr Spertus JA Costa F Diagnosis and management of the metabolic syndrome: an American Heart Association/National Heart, Lung, and Blood Institute Scientific Statement Circulation 2005 112 2735 2752 10.1161/CIRCULATIONAHA.105.169404 16157765

[B24] Ruan X Jin J Hua L Liu Y Wang J Liu S The prevalence of metabolic syndrome in Chinese postmenopausal women and the optimum body composition indices to predict it Menopause 2010 17 3 566 570 20054286 10.1097/gme.0b013e3181c8f4e1

[B25] Shah D The Annual conference of the British Menopause Society J Mid Life Health 2010 1 48 50 10.4103/0976-7800.66983 PMC3139266 21799641

[B26] Figueiredo Neto JA Figuerêdo ED Barbosa JB Barbosa Fde F Costa GR Nina VJ Nina RV Metabolic syndrome and menopause: cross-sectional study in gynecology clinic Arq Bras Cardiol 2010 95 339 345 10.1590/S0066-782X2010005000094 20658092

[B27] Feng Y Hong X Wilker E Li Z Zhang W Jin D Liu X Zang T Xu X Xu X Effects of age at menarche, reproductive years, and menopause on metabolic risk factors for cardiovascular diseases Atherosclerosis 2008 196 590 597 10.1016/j.atherosclerosis.2007.06.016 17675039 PMC2271121

[B28] Man KH Jong P Yeon R Jongoh K The effect of menopause on the metabolic syndrome among Korean women Diabetes Care 2007 30 701 706 10.2337/dc06-1400 17327344

[B29] Razy G Heaton KW Bolton CH Coronary heart disease risk factors in relation to the menopause Q J Med 1992 85 2–3 889 896 1484951

